# Actinomyces‐infected bronchopulmonary sequestration: An uncommon pathogen in a rare anomaly

**DOI:** 10.1002/ccr3.8984

**Published:** 2024-06-05

**Authors:** Aasir M. Suliman, Ahmed Alsayed, Sarah Obiedat, Ehab Massad, Irfan Ul Haq

**Affiliations:** ^1^ Pulmonology Department Hamad General Hospital, Hamad Medical Corporation Doha Qatar; ^2^ Laboratory Medicine and Pathology Department Hamad General Hospital, Hamad Medical Corporation Doha Qatar; ^3^ Thoracic Surgery Department Hamad General Hospital, Hamad Medical Corporation Doha Qatar

**Keywords:** Actinomyces, bronchopulmonary sequestration, case report, congenital lung anomaly, pulmonary actinomycosis

## Abstract

Bronchopulmonary sequestration, a rare congenital anomaly, involves a nonfunctioning lung tissue mass supplied by anomalous vessels. It is rarely infected by Actinomyces, further complicating the clinical presentation, with limited reported cases. This case highlights the distinctive clinical aspects, diagnostic challenges, and successful management strategies of such a rare clinical entity.

## 1 BACKGROUND

Bronchopulmonary sequestration (BPS) describes a nonfunctional dysplastic lung tissue forming a segment or lobe that develops without communication with the rest of the tracheobronchial tree system. This dysplastic lung tissue is fed by an isolated anomalous systemic vascular supply.^1^ BPS usually appears over lower lobes and divides into intralobar and extralobar sequestration. It presents a diagnostic challenge, often necessitating surgical intervention for definitive diagnosis and treatment.^2^ While BPS is infrequent, its association with actinomycosis further complicates the clinical presentation, rendering it an even rarer clinical entity.

Actinomyces is a filamentous, gram‐positive anaerobic bacterium that is typically commensal in the oral, gastrointestinal, and female genital tract flora.^3^ All body tissues can be affected, with a distinguished four main clinical types of infection: cervicofacial, thoracic, abdominopelvic, and disseminated disease.^4^ Cervicofacial actinomycosis represents the most common site of infection.^5^ Thoracic actinomycosis results from aspiration of the oropharyngeal or gastrointestinal tract secretions into the lung.^6^ Antibiotic therapy should be the mainstay treatment of actinomycosis, with surgery as a supporting therapy in refractory cases.^3^

This case report describes a unique clinical presentation of BPS complicated by Actinomyces infection. We present a case of a 48‐year‐old man who presented with a two‐month history of productive cough and hemoptysis, culminating in a life‐threatening episode of massive hemoptysis during admission. Urgent computed tomography (CT) chest imaging and subsequent surgical intervention were crucial for diagnosis and prompt management. Actinomyces infection within the context of BPS is exceedingly unusual, with limited cases reported in the literature.

## 2 CASE HISTORY/EXAMINATION

A 48‐year‐old man with a past medical history of Type 2 Diabetes Mellitus was referred to our facility with chronic cough and hemoptysis of 2 months duration. The cough was progressive, productive, and associated with a nonprogressive small amount (teaspoon) of hemoptysis for the same duration. History was also remarkable for unintentional weight loss of 13 kg over the last 3 months prior to presentation. A review of the other systems was negative for fever, night sweats, shortness of breath, chest pain, and fatigue. There was no history of sick contact or recent travel. Oral hypoglycemic agents were the only medications used by him. His family history was noncontributory; he drank liquor 1–2 times monthly and had a 10‐pack‐year smoking history. Physical examination was only significant for finger clubbing and right‐sided basal crackles on lung auscultation. He was on average build (body mass index of 22) and had a good oral dentition with no other remarkable findings on the remaining physical examination.

## 3 DIFFERENTIAL DIAGNOSIS, INVESTIGATIONS AND TREATMENT

**FIGURE 1:**
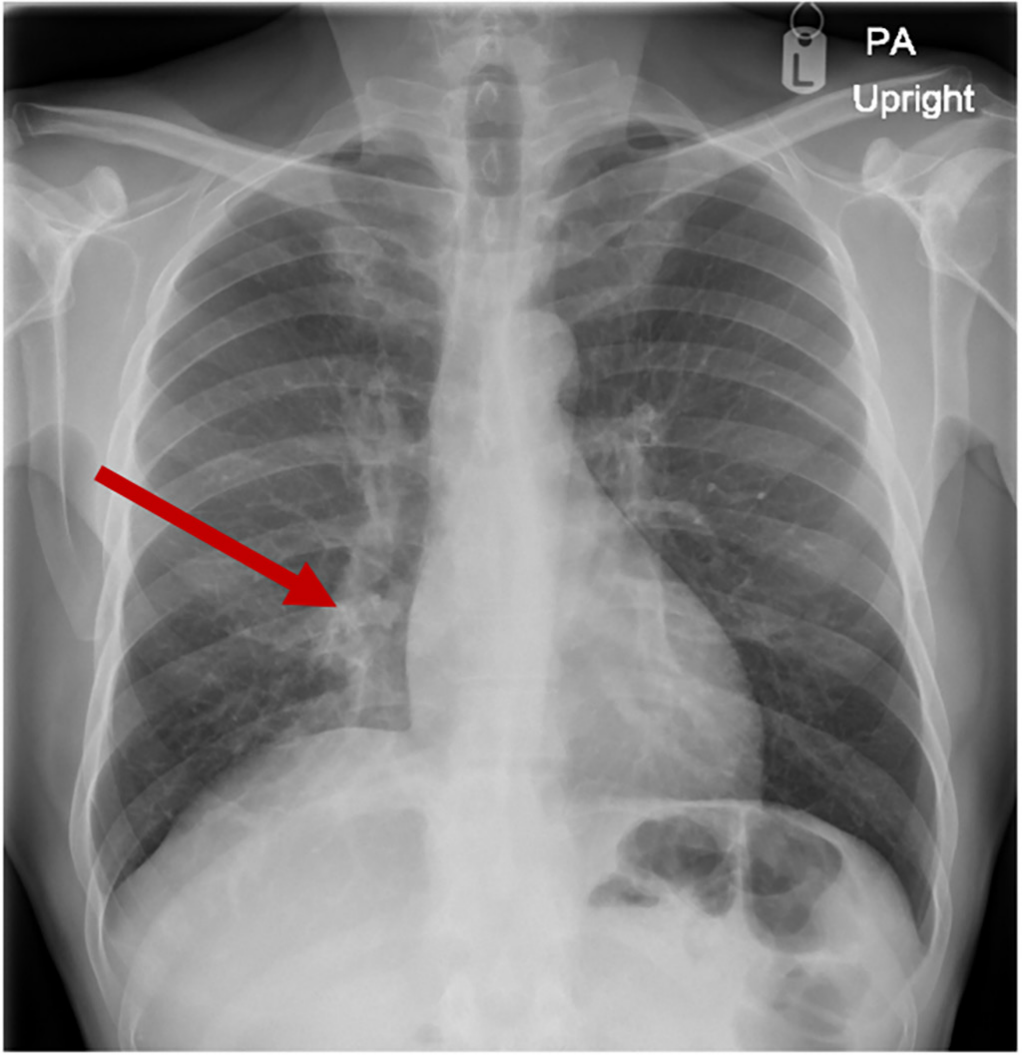
A chest XR showing ill‐defined opacity at the right cardio‐phrenic angle (Red arrow).

Initial laboratory workup showed white blood cell count (WBC) of 10.4 × 10^3^/μL (reference range 4–10 × 10^3^/μL), hemoglobin of 11.4 gm/dL (reference range 13–17 gm/dL) with features of microcytosis, mildly reduced albumin of 32 gm/L (reference range 35–50 gm/L), C‐reactive protein (CRP) of 62.2 mg/L, and high hemoglobin A1C of 10.5%. Other laboratory investigations including coagulation profile, renal function, and liver enzymes were all within normal limits. A chest radiograph (Chest XR) showed an ill‐defined opacity at the right cardiophrenic angle region (shown in Figure 1). Accordingly, the patient was admitted as a case of suspected pulmonary tuberculosis (TB). Sputum samples were sent for acid‐fast bacilli smear, PCR, and culture; all results were later reported as negative.

**FIGURE 2:**
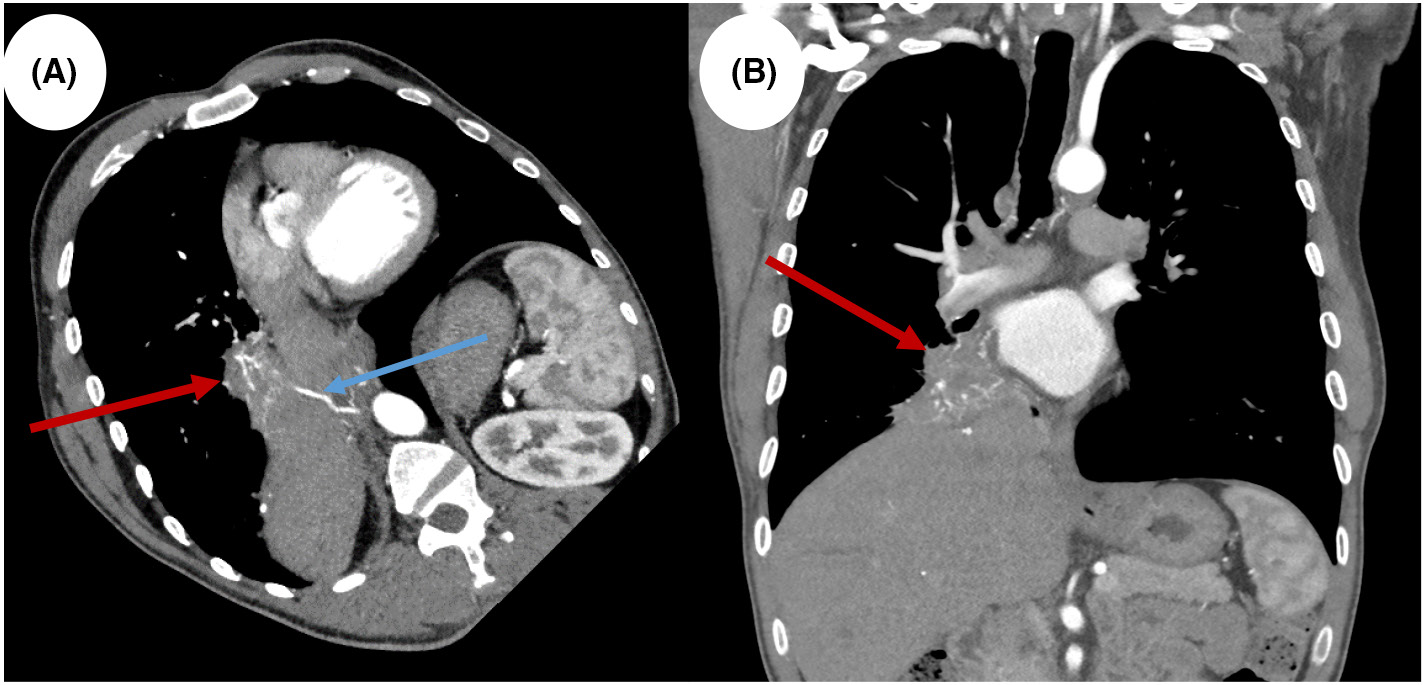
Oblique reconstruction (A) and coronal (B) computed tomography views demonstrate a large irregular heterogenous lesion in the inferior and medial aspect of the right lower lobe (red arrows), measures about 7.4 × 4.6 × 6 cm as anteroposterior (AP), transverse (TR), and craniocaudal (CC) diameters respectively with an anomalous arterial supply (blue arrow).

**FIGURE 3:**
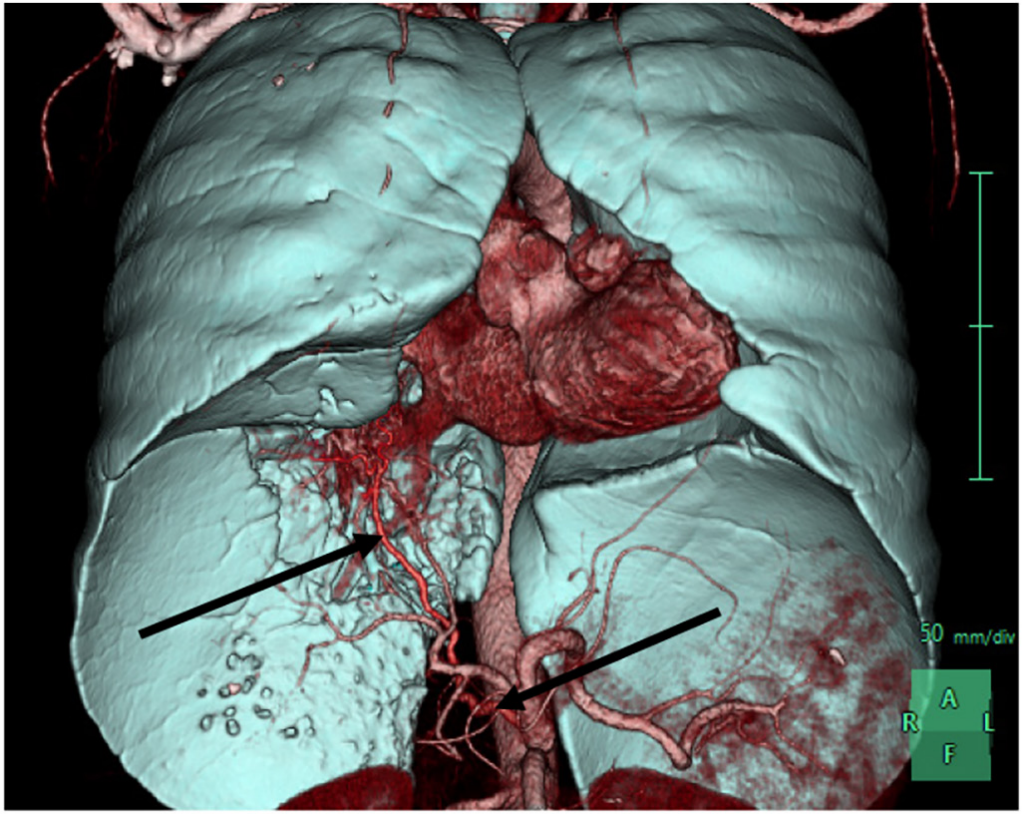
3D volume rendered image showing anomalous artery originating from the coeliac trunk supplying the sequestrated lung lesion (black arrows).

The patient experienced a frank hemoptysis episode on the second day of hospitalization, resulting in 20–30 mL of fresh blood with blood clots. His vitals were stable during the episode. An urgent computed tomography angiogram of the pulmonary vessels (CTPA) showed a large irregular heterogeneous lesion located at the inferior and medial aspect of the right lower lobe (shown in Figure 2). The lesion had a marked vascularity with arterial supply arising from the coeliac trunk and venous drainage to the right pulmonary vein (shown in Figure 3).

**FIGURE 4:**
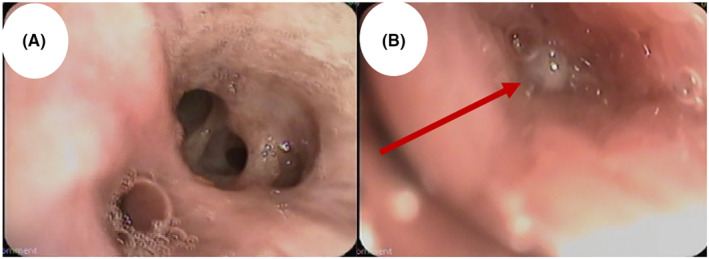
An endobronchial view during flexible bronchoscopy shows the right lower lobe (A) with an endobronchial lesion obscuring the medial segment of the right lower lobe (B) (red arrow).

A subsequent flexible bronchoscopy showed a protruding endobronchial lesion at the medial segment of the lower lobe (shown in Figure 4). Multiple Broncho alveolar lavages and endobronchial biopsies were taken from the same area, and they were all negative for infectious pathogens (gram stain and culture, acid‐fast bacilli culture, and fungal culture) or malignant cells (cytology and histopathology). As a result, the patient was referred to the Thoracic surgery team, and planned for a right lower lobectomy after a pulmonary function test that showed a good lung reserve.

Intraoperatively, the right lung was adherent to the chest wall with a tough fibrous adhesion. There was a hard mass‐like feeling in the right lower lobe with a minimal inflammatory process in the right middle lobe. A specimen measuring 14 × 10 × 8 cm and weighing 465 g was obtained. Gross sectioning revealed an irregular, firm tan area with central yellow pus measuring 6.4 × 4.9 × 3.1 cm. The lesion was 1 cm away from the bronchial and vascular margins, 3.8 cm from the parenchymal margin, and abutting the pleura.

**FIGURE 5:**
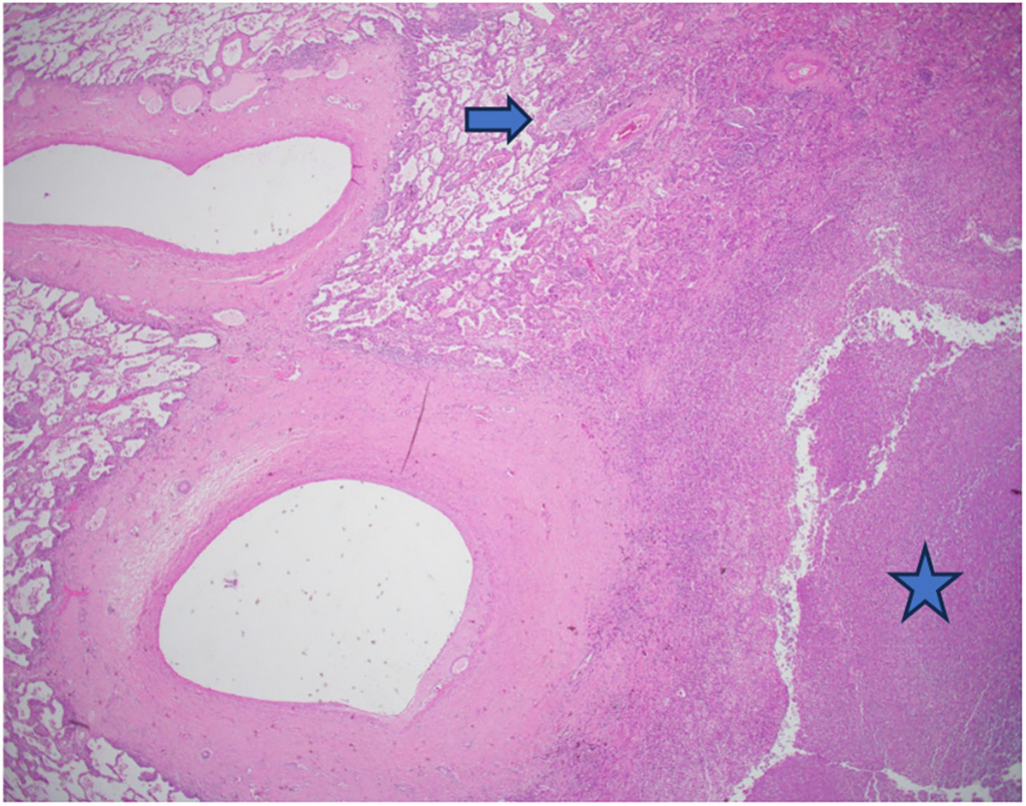
Large, thickened blood vessels with adjacent area of abscess formation (star). fibroblastic plugs or Masson bodies are seen in alveolar sacs or ducts (arrow).

**FIGURE 6:**
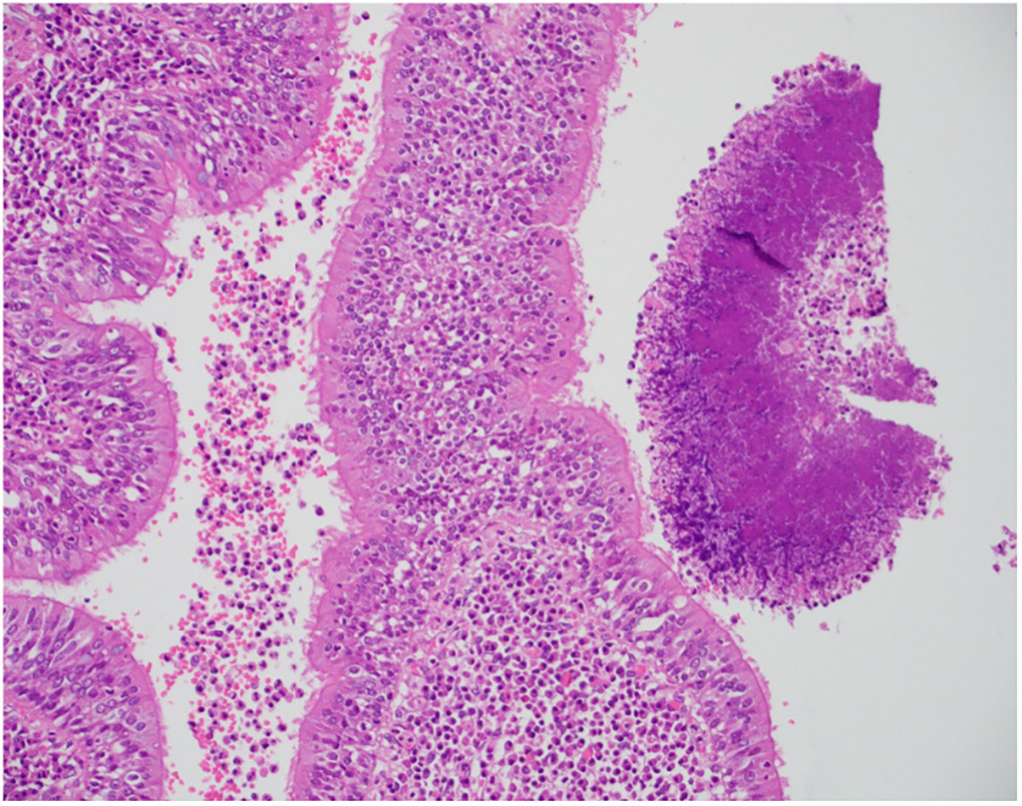
Medium power view of a colony of Actinomyces species showing filaments radiating from a central mass within a dilated and inflamed bronchiole.

Microscopic findings revealed lung parenchyma heavily infiltrated by acute and chronic inflammatory cells constituting both neutrophils and macrophages with abscess formation and surrounding lymphoid follicles. Masson bodies were identified (shown in Figure 5). Actinomyces microorganisms with eosinophilic radiating filamentous material resembling Splendore–Hoeppli phenomenon were seen within lung bronchioles and parenchyma (shown in Figure 6). There was no evidence of granulomas, dysplasia, or malignancy. Histopathology examination concluded a diagnosis of BPS associated with actinomycosis within lung abscess.

## 4 OUTCOME AND FOLLOW‐UP

The patient was started on a 90‐day treatment course for actinomycosis, including oral amoxicillin 1000 mg every 8 h, and discharged on Day 8 post surgery with an outpatient Thoracic surgery and Infectious disease clinic follow‐up. On several subsequent follow‐ups, the patient reported a significant improvement in his cough and a complete resolution of hemoptysis without any remarkable radiological findings.

## 5 DISCUSSION

Bronchopulmonary sequestration is rare, constituting about 1%–6% of all congenital lung anomalies, and it can often remain undetected during the prenatal period and early childhood years, exemplified in our case, who presented at the age of 48 years old.^7^ It consists of a nonfunctional segment or lobe of dysplastic lung tissue that does not communicate with the rest of the tracheobronchial tree and receives an anomalous vascular supply.^1^ Interestingly, in a retrospective analysis of 2625 cases of BPS, the aorta was the main supplying vessel in more than 94% of cases, whereas in one case only, the celiac artery was found to be the feeding vessel, similar to our patient.^8^ Apart from incidental diagnosis on chest imaging, the most common clinical presentation of intralobular sequestration is recurrent pneumonia.^9^ It can also present with hemoptysis; however, massive hemoptysis, similar to our case, is not frequently reported. The gold standard for identifying BPS is computed tomography/magnetic resonant angiography (CTA/MRA), as it confirms the anatomy, identifies the anomalous systemic arterial supply, and shows venous drainage.^8^ The management of BPS remains multidisciplinary, with surgical resection being the current standard and curative treatment, even in asymptomatic cases, to mitigate the risk of recurrent infection.^10^

While numerous patients with BPS may remain asymptomatic, the insufficient drainage caused by limited communication with healthy lung tissue creates favorable conditions for bacterial growth and serves as a nidus for future recurrent infections.^8^ The most commonly reported organisms in cases of BPS superinfection are *Streptococcus pneumoniae*, *Haemophilus influenzae*, and *Staphylococcus aureus*; However, other uncommon organisms like Nocardia, nontuberculous mycobacteria, Aspergillus, and Mycobacterium tuberculosis have been reported as well.^6^ The noteworthy aspect of this case is the association of Actinomyces infection with BPS, which is exceedingly rare, and up to our knowledge, there are only two reported cases so far.^6^, ^11^

Actinomycosis is a rare, chronic granulomatous infection caused by the bacterial species Actinomyces; although it can develop at various sites in the human body, pulmonary actinomycosis is relatively uncommon (15%).^12^ Conditions linked to aspiration, like alcohol consumption, are the main risk factors for pulmonary actinomycosis, whereas poor dental hygiene and diabetes predispose individuals more to cervicofacial actinomycosis.^15^ Clinically, Pulmonary actinomycosis is often nonspecific and confused with other chronic suppurative lung diseases and with malignancy, as was the case with our patient who was initially admitted for suspected pulmonary tuberculosis.^13^ A definitive diagnosis of actinomycosis typically involves isolating the pathogen from the affected organs, and this is due to the challenging nature of isolating this anaerobic bacterium from clinical specimens.^14^ Pulmonary actinomycosis is typically treated with an extended regimen of beta‐lactam antimicrobials for a total duration of 2–12 months depending on the disease severity.^13^ Although surgical intervention is not always necessary, the unusual association of BPS with actinomycosis such as in our case, rendered it crucial.

In conclusion, this case report highlights the rare association between BPS and pulmonary actinomycosis. Due to their similar nonspecific clinical presentation, early identification of this complex entity can be challenging, with the suboptimal response to standard antimicrobials as a potential clue. Accurate identification of the causative organism, especially atypical pathogens like Actinomyces, is crucial in BPS patients, given their higher infection incidence. Our patient's significant clinical improvement and positive outcome emphasize the importance of timely, accurate, and efficient diagnosis for this atypical clinical entity.

## AUTHOR CONTRIBUTIONS

**Aasir M. Suliman:** Conceptualization; writing – original draft; writing – review and editing. **Ahmed Alsayed:** Writing – original draft. **Sarah Obiedat:** Investigation. **Ehab Massad:** Investigation. **Irfan Ul Haq:** Writing – review and editing.

## FUNDING INFORMATION

This article is funded by the Qatar National Library whom they have no role in manuscript writing.

## CONFLICT OF INTEREST STATEMENT

The authors declare that they have no known competing financial interests or personal relationships that could have appeared to influence the work reported in this paper.

## ETHICS STATEMENT

The case report is approved by the Medical Research Centre at Hamad Medical Corporation and the Hamad Institutional Review Board (IRB) under number MRC‐04‐23‐779.

## CONSENT

Written informed consent was obtained from the patient to publish this report in accordance with the journal's patient consent policy.

## Data Availability

The data sets used and/or analyzed during the current study are available from the corresponding author upon reasonable request.
